# Eco-evolutionary trade-offs in the dynamics of prion strain competition

**DOI:** 10.1098/rspb.2023.0905

**Published:** 2023-07-12

**Authors:** Saul Acevedo, Alexander J. Stewart

**Affiliations:** ^1^ Department of Biology, University of Houston, Houston, TX, USA; ^2^ School of Mathematics and Statistics, University of St Andrews, St Andrews KY16 9SS, UK

**Keywords:** prions, eco-evolutionary trade-offs, adaptive dynamics

## Abstract

Prion and prion-like molecules are a type of self-replicating aggregate protein that have been implicated in a variety of neurodegenerative diseases. Over recent decades, the molecular dynamics of prions have been characterized both empirically and through mathematical models, providing insights into the epidemiology of prion diseases and the impact of prions on the evolution of cellular processes. At the same time, a variety of evidence indicates that prions are themselves capable of a form of evolution, in which changes to their structure that impact their rate of growth or fragmentation are replicated, making such changes subject to natural selection. Here we study the role of such selection in shaping the characteristics of prions under the nucleated polymerization model (NPM). We show that fragmentation rates evolve to an evolutionary stable value which balances rapid reproduction of *PrP*^*Sc*^ aggregates with the need to produce stable polymers. We further show that this evolved fragmentation rate differs in general from the rate that optimizes transmission between cells. We find that under the NPM, prions that are both evolutionary stable and optimized for transmission have a characteristic length of three times the critical length below which they become unstable. Finally, we study the dynamics of inter-cellular competition between strains, and show that the eco-evolutionary trade-off between intra- and inter-cellular competition favours coexistence.

## 1. Introduction

Prions are a well-known class of aggregative proteins responsible for a number of neurodegenerative disorders, including Creutzfeldt–Jakob disease (CJD) in humans, bovine spongiform encephalopathy (BSE) in cattle and scrapie in sheep. Illnesses is caused by misfolding of the PrP protein (*PrP*^*Sc*^), which coerces the benign conformation (*PrP*^*c*^) into an abnormal state. The misfolded *PrP*^*Sc*^ then aggregates within the brain, forming transmissible spongiform encephalopathies (TSEs) [1], which impair brain function and ultimately result in death. As a result, the biochemistry of prion replication has been studied thoroughly, and the molecular dynamics have even been characterized mathematically via the nucleated polymerization model (NPM) [2–8].

The role of natural selection in shaping the biochemistry of prion strains has become increasingly clear over recent decades [9–12]. Strains accumulate ‘mutations’ in their conformation which alter their rate of reproduction (in which a prion aggregate splits into two separate viable aggregates of shorter length), by changing the rate at which they co-opt *PrP*^*c*^ into their aggregate, for example, or the rate at which they fragment into multiple aggregates sharing the biochemical properties of the parent. The result is replication coupled with heritable variation, which provides the basis for a form of Darwinian evolution in which the protein aggregates themselves are the basic unit of replication. The question that immediately arises is: what kind of prions will this natural selection produce?

We answer this question by adapting the NPM to study the evolutionary trade-offs faced by prion strains. We characterize the dynamics of inter-and intracellular competition between strains [13,14], and show that, in a well-mixed environment, selection acts to produce strains that most efficiently exploit the *PrP*^*c*^ resource produced by a cell. Next we show that when strains compete to invade new cells, selection produces an entirely different optimum, since strains must minimize their probability of extinction when rare. And so prion strains face a trade-off between their ability to infect new cells, and their ability to efficiently make use of the *PrP*^*c*^ resources within a cell.

In order to understand the consequences of this trade-off, we study the ecological dynamics of competing prion strains under a compartmental model of cell infection. We show that competition between strains that are optimized to infect new cells or to co-opt *PrP*^*c*^ within a cell, respectively, leads to coexistence, and in some circumstances to sustained oscillations with repeated waves of infection and reinfection. Our results show that competition between prion strains can generate complex dynamics, with potential consequences for the epidemiology of prion diseases, and for the eco-evolutionary dynamics of the amyloid world more generally.

## 2. Model

### (a) Intra-cellular model

We study eco-evolutionary competition between prion strains using models of intra- and inter-cellular prion dynamics. We focus on well-mixed models, and include a spatial model of inter-cellular competition in the electronic supplementary material. Our model of intra-cellular dynamics is based on the well-studied NPM [2–4]. The NPM assumes that two forms of the prion protein exist: the monomeric *PrP*^*c*^ conformation and the pathogenic aggregate form *PrP*^*Sc*^. Aggregates grow linearly, with existing aggregates converting monomers and incorporating them into the aggregate structure. Replication of an aggregate occurs when it fragments into two separate daughter aggregates. These dynamics are described via a system of ODEs which capture the time evolution of prion aggregates (both the number of individual aggregates and their average size) [2–4] as follows:

2.1 $$\begin{array}{cc} & \frac{dx}{dt}=\lambda -xd-\beta xy+n(n-1)\;by,\\ & \frac{dy}{dt}=-ay+bz-(2n-1)\;by\\ \mathrm{and}\; & \frac{dz}{dt}=\beta xy-az-n(n-1)\;by, \end{array}\}$$

where *x* is the abundance of monomeric *PrP*^*c*^, *y* is the abundance of individual *PrP*^*Sc*^ polymers and *z* is the total abundance of polymerized subunits. The rate parameters, *λ*, *β*, *b*, *a* and *d* describe the rate of monomer production, monomer to polymer conversion, aggregate fragmentation, polymer clearance and monomer degradation, respectively (table 1). The parameter *n* describes a critical size below which aggregates disintegrate into their constituent monomers.

**Table 1.** Intra- and inter-cellular model parameters.

parameter	definition	model
*a*	clearance rate of polymers	NPM
*β*	monomer to polymer conversion rate	NPM
*b*	fragmentation rate	NPM
*λ*	monomer production rate	NPM
*d*	monomer degradation rate	NPM
*n*	minimum polymer length (critical size)	NPM
*μ*	rate of mutations to *b*	NPM
*B*_1_	rate of strain 1 infecting uninfected cells	compartmental
*B*_2_	rate of strain 2 infecting uninfected cells	compartmental
*D*	rate of strain 1 infecting strain 2 cells	compartmental
*ν*_1_	death rate of strain 1 infected cells	compartmental
*ν*_2_	death rate of strain 2 infected cells	compartmental

In our study, we combine analysis of the ODE system equation (2.1) with stochastic simulations based on the NPM. Under the stochastic NPM, we study the number of monomers *X* and the number aggregate polymers of length *i* *Y*_*i*_. Monomers are produced by the cell (*X* → *X* + 1) at rate *λ* and degraded (*X* → *X* − 1) at rate *d*. Similarly, a *PrP*^*Sc*^ aggregate is extended by one monomer unit (*Y*_*i*_ → *Y*_*i*−1_, *Y*_*i*+1_ → *Y*_*i*+1_ + 1) with rate *β*. Growth of the aggregate by assimilation of a monomer results in *X* → *X* − 1. Each aggregate of size *i* has *i* − 1 links connecting its *PrP*^*c*^ subunits together, and each of them is assumed to fragment with fixed rate *b*. Therefore, an aggregate of size *i* fragments at rate *b*(*i* − 1) into two smaller daughter aggregates of length *j* and *i* − *j*. And so the size of the daughter cell, *j*, varies between a minimum of 1 and a maximum of *i* − 1. If the length of either of the daughters is below a critical size *n* (e.g. if one of the daughters has *j* < *n*), that prion disintegrates into *j* monomers. Finally, cellular degradation of individual aggregates (*Y*_*i*_ → *Y*_*i*−1_) occurs at rate *a*.

**Figure 1.**
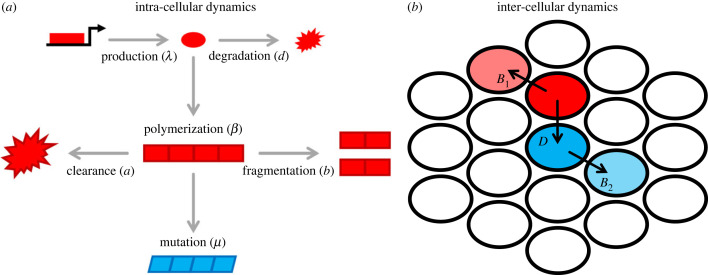
Intra- and inter-cellular prion dynamics: (*a*) Within a cell, we adapt the nucleated polymerization model (NPM) to describe the evolution of prion strains. We model the molecular dynamics of prions as a Markov process with transitions in which aggregates undergo the processes of polymerization at rate *β*, fragmentation at rate *b*, degradation at rate *d* and clearance at rate *a*. We also assume that mutations can occur, at rate *μ* in which the fragmentation rate *b*_*k*_ of a polymer *k* is perturbed by an amount Δ compared to its parent. This represents the emergence of a new strain (blue) from a resident strain (red). (*b*) We model the dynamics of inter-cellular competition via a compartmental model in which two strains *P*_1_ (red) and *P*_2_ (blue) invade uninfected cells (white) at rates *B*_1_ and *B*_2_, respectively. We also assume that the first strain can invade the second strain, which occurs at rate *D*.

The primary focus of this paper is the *evolution* of prion aggregates, in which spontaneous ‘mutations’ occur in the form of conformation changes, which alter the biochemical properties of the prion in a way that is sustained across subsequent fragmentation/reproduction events (figure 1). We focus in particular on the evolution of fragmentation rates, *b*. We assume that changes in the fragmentation rate of a daughter aggregate occur with rate *μ* at each fragmentation/reproduction event. Mutations are assumed to have small effects (i.e. a mutated daughter is assumed to have a new fragmentation rate that is a perturbation about the aggregation rate of the mother). We assume that mutation sizes are normally distributed with mean 0 and variance 0.01. Thus, different prion aggregates can have different fragmentation rates, corresponding to different ‘strains’. In the electronic supplementary material, we also discuss evolution of other prion ‘traits’: the clearance rate *a* and the growth rate *β* of *PrP*^*Sc*^ aggregates within a cell. The model parameters for both the inter- and intra-cellular models are summarized in table 1.

### (b) Inter-cellular model

In order to study inter-cellular prion dynamics, we first analyse the probability of *invasion* of an uninfected cell by a single prion under the NPM. We distinguish this from the rate of *transmission*, which is the rate at which prions pass between cells. Thus the rate of *infection* of previously uninfected cells by a given prion strain depends on the rate of transmission multiplied by the probability of invasion:

$$\begin{array}{rl} \text{rate of infection} & =\text{rate of transmission}\\ & \;\times \text{probability of invasion}. \end{array}$$

Thus, understanding the probability of invasion is crucial for understanding inter-cellular prion dynamics.

Having analysed the probability of invasion under the NPM, we develop a compartmental model of inter-cellular prion dynamics in which the rates of infection of cells reflect the insights gained from our analysis of the NPM. Under this model, two prion strains 1 and 2 compete to infect cells. The strains invade and infect previously uninfected cells at rate *B*_1_ and *B*_2_, respectively (reflecting their different success at invasion), while strain 1 is able to infect strain 2 at rate *D* (reflecting intra-cellular prion competition, figure 1). Cells die at rate *ν*_1_ and *ν*_2_, respectively. The resulting compartmental model is as follows:

2.2 $$\begin{array}{cc} & \frac{dS}{dt}=S(1-S)-S(B_{1}P_{1}+B_{2}P_{2}),\\ & \frac{dP_{1}}{dt}=B_{1}SP_{1}+P_{I}P_{2}D-\nu_{1}P_{1}\\ \mathrm{and}\; & \frac{dP_{2}}{dt}=B_{2}SP_{2}-P_{I}P_{2}D-\nu_{2}P_{2}, \end{array}\}$$

where uninfected cells have density *S* and are assumed to undergo logistic growth, while cells infected by prion strains 1 and 2 have density *P*_1_ and *P*_2_, respectively (table 1), where all densities are normalized with respect to the carrying capacity of uninfected cells in the absence of any infection.

## 3. Results

### (a) Prion evolution

We begin by studying evolution of the prion fragmentation rate *b*. Evolution of a prion trait is said to occur if perturbations to the structure of a prion aggregate, which alter the trait value, is passed on to its daughters following fragmentation. Since such a change would alter the birth and/or death rate of the daughter prion aggregates, such a change corresponds to heritable variation and provides the basis for a form of evolution. We can analyse the evolutionary dynamics of these traits by adopting an approach similar to adaptive dynamics [15], in which we assess the growth rate of a rare new strain which is perturbed from the resident strain by a small amount.

We show (see electronic supplementary material, section S1) that under the NPM, any mutant strain which decreases the equilibrium amount of free monomer *PrP*^*c*^ is able to invade and replace a resident strain. And so the evolutionary dynamics of prion aggregates lead to optimal use of the resource *PrP*^*c*^. Considering the parameters (*b*, *a*, *β*), individually, we find that increasing accumulation rate *β* and decreasing clearance rate *a* will always evolve (since they produce larger, longer lived prion aggregates and so reduce the overall death rate of the strain). And so these parameters are constrained to their physical maximum and minimum, respectively, by the process of natural selection. The fragmentation rate *b*, in contrast, has an evolutionary optimum. Since more rapid fragmentation increases the rate of prion reproduction (if the resulting fragments exceed the critical length *n*) but also the rate of prion death (since if prions are too short when they fragment, both offspring may be shorter than the critical length *n*, resulting in both being removed from the population), there is a trade-off. We find that the evolutionarily stable fragmentation rate *b*_opt_ is given by

**Figure 2.**
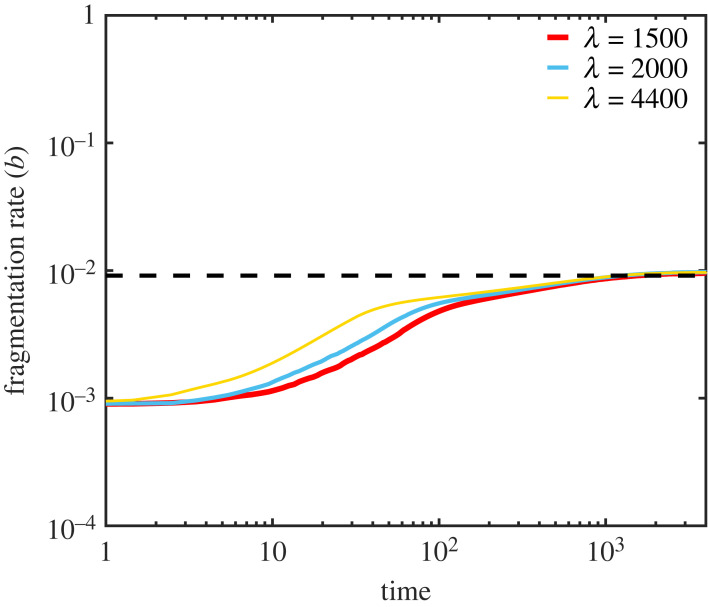
The evolution of fragmentation rate: Prion fragmentation rate evolves to the evolutionary optimum. We simulated the stochastic NPM using the Gillespie algorithm [16,17]. When evolution of fragmentation rate *b* is allowed the system reaches the evolutionary optimum *b*_opt_ for different values of *λ*. Parameters used are *n* = 6, *a* = 0.05, *β* = 0.015, *d* = 4 and *μ* = 0.01. Simulation data are an average of 1000 simulations.

3.1 $$b_{\mathrm{opt}}=\frac{a}{\sqrt{n(n-1)}}$$

which depends only on the prion clearance rate *a* and the critical length *n*. We simulated prion evolution using the stochastic NPM described above (figure 2) and show that strains do indeed evolve to the optimum fragmentation rateequation (3.1), in a manner that does not depend on the rate of monomer production.

**Figure 3.**
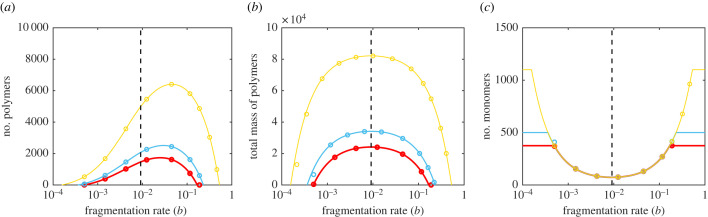
Prion abundance. (*a*) The fragmentation rate that maximizes the equilibrium number of *PrP*^*Sc*^ aggregates for both simulations (points) and the deterministic NPM equilibrium (lines) does not coincide with the evolutionarily stable fragmentation rate (vertical dashed line). (*b*) The fragmentation rate that maximizes the equilibrium number of *PrP*^*Sc*^ subunits contained in aggregates coincides with the evolutionarily stable fragmentation rate as predicted. (*c*) Similarly, the fragmentation rate that minimizes the equilibrium number of *PrP*^*c*^ monomers coincides with the evolutionarily stable fragmentation rate as predicted. We simulated the stochastic NPM using the Gillespie algorithm [16,17]. Parameters and colours are as indicated in figure 2. Simulation data are an average of 1000 simulations.

Having shown that natural selection acts to maximize exploitation of *PrP*^*c*^ by *PrP*^*Sc*^, we explored the kinds of prion that this process would produce (figure 3). Naively, we might expect natural selection to maximize the number of individual *PrP*^*Sc*^ aggregates, however previous work has shown [6] that, when strains compete, the dominant strain will be that which minimizes the amount of free monomer. We show that evolution proceeds on the same principle (see electronic supplementary material, section S1).

Figure 3 shows how the number of aggregates, the total mass of aggregates and the number of free monomers vary with fragmentation rate *b*. We see that while the optimum fragmentation rate does not maximize the number of *PrP*^*Sc*^ aggregates, it simultaneously maximizes the total mass of protein contained in *PrP*^*Sc*^ while minimizing the number of free monomers (see also electronic supplementary material, section S1). And so we can view natural selection as maximizing the total number of individual *PrP*^*Sc*^ proteins.

### (b) Optimizing prion invasion

Our results on the evolution of prion traits have so far been constrained to competition between strains within a cell however as with any epidemiological phenomenon, we must also consider the dynamics of infection. As described above, we study prion infection as comprising both a transmission event, in which a prion passes from one cell to another, followed by an invasion event, in which a single prion successfully establishes a population within a cell. We can study the probability of invasion of a single *PrP*^*Sc*^ aggregate into a previously uninfected cell analytically (see electronic supplementary material, section S2). We show that the optimal rate of *invasion* $b^{†}$ can be approximated by

3.2 $$b^{†}\approx \frac{\beta \lambda }{d}\frac{1}{{\left(2n-1\right)}^{2}}.$$

**Figure 4.**
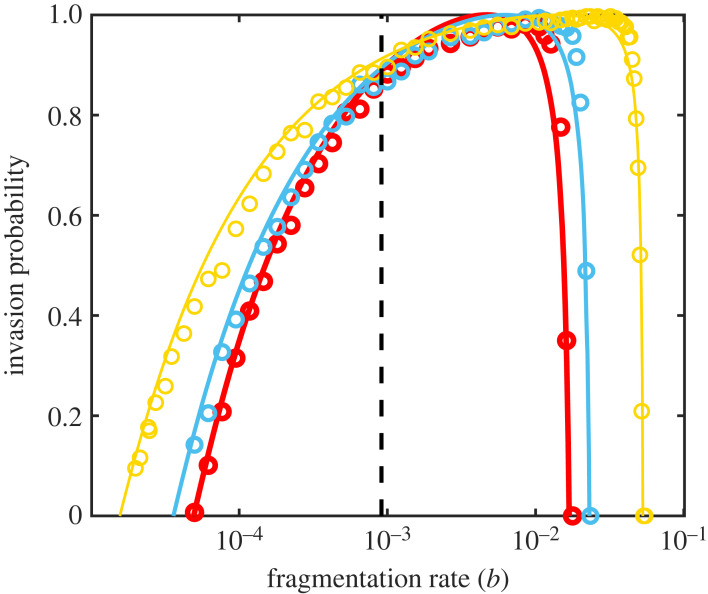
Invasion of uninfected cells: We simulated the stochastic NPM using the Gillespie [16,17] algorithm, initializing each simulation with a single *PrP*^*Sc*^ aggregate drawn from the equilibrium distribution (points) and compared the results to the analytical invasion probability (see electronic supplementary material, section S2). We see that the fragmentation rate that maximizes the invasion probability differs from the evolutionarily stable fragmentation rate (black dashed line). Parameters used are *n* = 6, *a* = 0.05, *β* = 0.015, *d* = 4. Other parameters are as indicated in figure 2. Simulation data are an average of 1000 simulations.

Assuming that transmission rate is the same for different prion strains, equation (3.2) can also be understood as the optimal infection rate. Equations (3.1) and (3.2) are not the same in general (figure 4), and indeed we show in the electronic supplementary material (section S2) that their ratio is approximated by

3.3 $$\frac{b^{†}}{b_{\mathrm{opt}}}\approx \frac{\beta \lambda }{4nda}.$$

Since intra-cellular natural selection tends to maximize *β* while minimizing *a*, we might expect the ratio equation (3.3) to differ significantly from 1, resulting in slow prion transmission when evolution acts on timescales that lead to optimization for intra-cellular competition. And so prions face an evolutionary trade-off analogous to the virulence-transmission trade-off faced by many pathogens [18,19].

As we have argued above, the other prion traits *a* and *β* evolve to their minimum and maximum, respectively, and so are determined by physical constraints. Equation (3.2) allows us to conclude that the most successful prion strains are those for which *βλ*/4*n da* ≈ 1, and so cannot be invaded by intra-cellular mutations but also maximize their rate of transmission. The characteristics of such a strain can be determined by calculating the average aggregate size $\bar{s}$. Such prions, which simultaneously optimize for inter- and intra-cellular competition, have average length $\bar{s}\approx 3n$ (see electronic supplementary material, section S2).

### (c) Ecological competition between strains

Having characterized the optimal fragmentation rate for intra-cellular competition and inter-cellular spread, we next consider ecological competition between prion strains. Our results show that there is a trade-off between the ability to successfully infect new cells, on the one hand, and the ability to displace another prion strain from an already infected cell, on the other. And so we focus our analysis on a scenario in which two competing strains, 1 and 2, differ such that the rate of infected susceptible cells is greater for strain 2 than strain 1, i.e. *B*_2_ > *B*_1_ (see equation (2.2)). However, the first strain dominates in intercellular competition, and invades cells already infected with strain 2 at rate *D*. This scenario captures the eco-evolutionary trade-off faced by prion strains under the NPM model.

**Figure 5.**
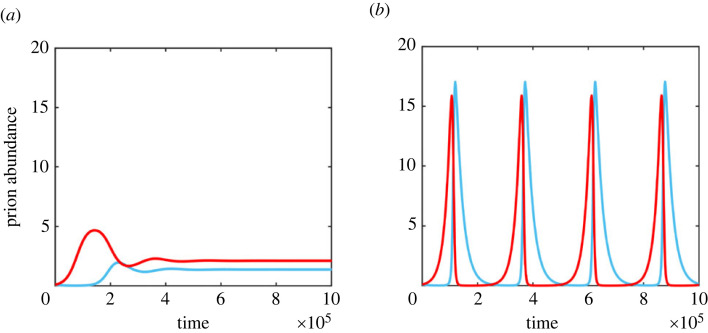
Intra-cellular competition. (*a*) The compartmental model of intra-cellular competition (equation (2.2)) produces stable coexistence when strain 1 (blue) can invade strain 2 (red) but strain 2 is better at invading uninfected cells than strain one, which occurs when the first strain has the evolutionarily stable fragmentation rate *b*_opt_ while the second has the optimal fragmentation rate for transmission $b^{†}$. (*b*) When uninfected cell dynamics are much faster than prion dynamics (see electronic supplementary material) the same process produces stable limit cycles, in which strain 2 invades, only to be replaced by strain 1, before both die out, in a pattern similar to Lotka–Volterra dynamics. Parameters used are *B*_1_ = 0.01, *B*_2_ = 0.1, *ν*_1_ = *ν*_2_ = 0.05 and *D* = 0.02.

We show that under this scenario stable coexistence of stains either at a constant abundance or as limit cycles (figure 5) occurs, where limit cycles require that the dynamics of uninfected cells are much faster than the dynamics of prion infection (see electronic supplementary material, section S3). Note that when this trade-off is not present (i.e. when one strain is both worse at infecting and loses out in inter-cellular competition) coexistence will not occur.

Finally, we explore the dynamics of prion-strain coexistence under a spatial lattice model, and show that similar dynamics occur, with damped oscillations leading to stable coexistence between strains (see electronic supplementary material, figure S1). The spatial lattice model allows us to also see the damage done by prions through the empty regions left following cell death. Future research using this type of model may study the time course of prion diseases in more complex spatial environments.

## 4. Discussion

Prion and prion-like molecules have been studied for decades [20], both because of their role in neurodegenerative disease and, more recently for their role in shaping the evolutionary dynamics of the organisms they infect [21–23] and as the target of natural selection themselves [9–12]. Our results show that natural selection will act, in a well-mixed, intra-cellular environment, to optimize the exploitation of *PrP*^*c*^ by *PrP*^*Sc*^. However, we also show that the ability to successfully infect other cells leads to a different optimum, and so prions face an eco-evolutionary trade-off analogous to the virulence-transmissibility trade-off faced by many pathogens [18,19]. We then show that the existence of this trade-off implies that inter-cellular competition between strains implies coexistence between prion strains.

A key assumption of our evolutionary analysis is that ‘mutations’ that lead to conformational changes in prion aggregates are transmissible across fragmentation events. This kind of evolution involves no genetic component, and the extent to which our analysis is valid depends crucially on the biochemical properties of aggregates. Empirical studies of protein-encoded inheritance [24,25] has shown that transmissible polymorphism can be generated through alternative packing arrangements of *β*-sheets, thus providing the raw material for evolution. Nonetheless, this type of polymorphism is unlikely to satisfy the assumptions of a continuous spectrum of local mutations assumed in our analysis. However, a number of studies have shown a wide diversity of prion strains exist with conformations that are stable through replication [26–29], and undergo convergence to an adapted state [27]. More generally, the idea that evolutionary dynamics are shaped by biochemical factors which constrain the availability of mutations is well established in the study of genetic evolution [30] and the ‘mutational’ landscape of prion aggregates is an important direction for future empirical study.

Our results hold for the nucleated polymerization model (NPM) of prion dynamics, and as such are a simplified and idealized description of a more complex molecular process. We show that even under such a simplified model, natural selection produces complex and counterintuitive results. Our prediction that the most successful prion strains will have a characteristic size of roughly three times their critical size *n*. Although it is not clear that evolution will necessarily produce prions with these characteristics, where the combination of natural selection and physical constraints produce such molecules they will be both stable and able to spread. Our compartmental and spatial-lattice models of inter-cellular prion dynamics captures the trade-off between transmissibility and intra-cellular competition, and shows that it leads to coexistence between strains at the level of cells. This is in contrast to strain coexistence in a well-mixed environment (i.e. within a cell) which is not possible under the NPM [31] (see also electronic supplementary material, section 1.2) although can be seen in modified versions of the model such as the template assistance model can permit limited forms of intra-cellular coexistence. The compartmental model of inter-cellular prion dynamics also permits limit cycles in the limit that infection dynamics are slow compared to the dynamics of uninfected cell growth and death. This is unrealistic in the context of brain cells, where prion diseases have been identified, but may be relevant in the broader context of amyloids.

The NPM is a well studied, but highly simplified model of prion dynamics, in particular the assumptions about a critical length *n*. The model has been generalized in a number of ways [6–8,31], including to account for strains recruiting multiple monomer types [7]. Our detailed prediction, that the most successful prion strain will be such that its average length is three times its critical length clearly depends on the validity of the NPM for describing prion dynamics and therefore suggests a way of testing the assumption of the NPM empirically. However, we suggest that our qualitative conclusion—that prions face a trade-off between inter- and intra-cellular competition, and that this trade-off can be resolved for certain combinations of parameters, is likely to hold more generally.

Prions are a subclass of amyloids, a group of aggregative proteins that are characterized by a fibrillar, *β*-sheet rich structure [1,32,33]. Many amyloids are not pathogenic and provide essential functions [34,35]. However, amyloids also comprise other prion-like proteins called prionoids, responsible for a number of neurodegenerative diseases [1,32,33], such as amyloid-*β* which has been implicated in the development of Alzheimer’s disease [36–38]. The key distinction between prions and prionoids is that prionoids are not transmissible between individuals. And so understanding what determines the transmissibility of an amyloid has important practical implications. More generally, amyloids are understood as a source of primitive molecular replicators [39–41]. While it is generally accepted that RNA was the first self-replicating molecule, the high instability of RNA would probably have rendered it inactive in the extreme environment of the prebiotic soup [42]. By contrast, prions are quite stable and can persist in extreme environments, even at high temperatures [43]. Therefore, understanding the process of prion evolution could shed light on how self-replicating molecules could give rise to the first life.

Our work shows that the molecular dynamics of prion aggregates in the presence of heritable structural variation imply rich evolutionary dynamics via the trade-off between inter- and intra-cellular dynamics. We show that this trade-off is resolvable if prions have an average length of roughly three times their minimum length under the NPM. Under these circumstances, prions will be optimized for both infection and intra-cellular competition, and are therefore likely to be especially persistent. In general, however, prion evolution is subject to an eco- evolutionary trade-offs familiar from the study of other pathogens. The consequences of this trade-off depend critically on the space of replicable aggregate conformations accessible to evolution, which is an empirical question. We suggest that an empirical focus on the capacity of prions and prion-like protein aggregates to evolve is key to understanding their biological function and the degree of risk they pose as pathogens.

## Data Availability

The data are provided in electronic supplementary material [44].
